# Impact of social media on Working Memory and Academic Performance of Undergraduate Students- A Cross-sectional Study

**DOI:** 10.1192/j.eurpsy.2024.363

**Published:** 2024-08-27

**Authors:** N. E. Mgbedo, M. E. Musa, P. Chhikara, R. Chaturvedi, N. Vyas, N. Zavradashvili

**Affiliations:** ^1^Medicine, University of Georgia; ^2^Medicine, Batumi State University; ^3^Medicine, Eastern European University, Tbilisi, Georgia

## Abstract

**Introduction:**

Over the course of the last decade social media has become a very important part of the human experience; it has become the main source of communication and entertainment for a lot of people young and old.

**Objectives:**

This study aimed to examine the influence of social media on undergraduates working memory and academic performance. We assessed the association between the harmful use of social media on gender differences, physical activities, academic performance, and working memory.

**Methods:**

This cross-sectional study was designed to examine the effect of social media on the working memory of undergraduate students from three different Universities in Georgia involving 722 participants. The collection survey form was distributed among Undergraduate students from the University of Georgia (UG), Eastern European University (EEU), and Batumi State University (BSU) through google forms from 14th June to 2nd July 2023. The questionnaire consisted of sociodemographic characteristics (e.g., age, gender, and institution), social media disorder scale (SMD), academic performance scale (APS), and working memory (WM).

**Results:**

58.7% were female students, the mean age was 21.94 (SD ± 2.8), and most of the participants were international students. More students from Tbilisi had persistence (59.8%), escape (69%) complaints, and students from Batumi had more preoccupation (43%), persistence (62.5%) and escape (65.7%) complaints. 64.1% of female students are at increased risk of using social media as an ‘escape’ from negative feelings (OR 0.50; χ2 (18.206), p= 0.000, 95% Cl[0.368-0.692]). 51.6% of male students and 48.4% of female students had the risk of ‘conflict’ with families and friends because of social media (OR 1.65; χ2 (6.507), p= 0.011, 95% Cl[1.122-2.452]. 80.3% of students that had good academic performance are at risk of neglecting activities such as hobbies, sports, and class assignments because of social media (OR 0.63; χ2 (5.133), p= 0.023, 95% Cl[0.425-0.942]). 94% of students with good working memory had the risk of withdrawal complaints (OR 0.34; χ2 (6.865a), p= 0.009, 95% Cl[0.154-0.793]). As 93.4% of having conflicts with parents, siblings, and partners because of social media.

**Conclusions:**

Our studies presented the prevalence of social media addiction and its effect on academic performance and working memory among undergraduate students. The influence of social media on students has been significant. Students should establish boundaries, use digital moderation, and seek treatment for emotional difficulties as further studies are recommended.

**Disclosure of Interest:**

None Declared

